# Intestinal parasitic infections in children presenting with diarrhoea in outpatient and inpatient settings in an informal settlement of Nairobi, Kenya

**DOI:** 10.1186/1471-2334-13-243

**Published:** 2013-05-27

**Authors:** Cecilia Kathure Mbae, David James Nokes, Erastus Mulinge, Joyce Nyambura, Anthony Waruru, Samuel Kariuki

**Affiliations:** 1 Centre for Microbiology Research, P.O Box 19464–00202, Nairobi, Kenya; 2 KEMRI Wellcome Trust Research Programme, Kilifi, Kenya; 3 Kenya Medical Research Institute, Nairobi, Kenya

**Keywords:** Intestinal parasites, Urban slums, Children, Outpatients, Inpatients

## Abstract

**Background:**

The distribution of and factors associated with intestinal parasitic infections are poorly defined in high risk vulnerable populations such as urban slums in tropical sub-Saharan Africa.

**Methods:**

In a cross sectional study, children aged 5 years and below who presented with diarrhoea were recruited from selected outpatient clinics in Mukuru informal settlement, and from Mbagathi District hospital, Nairobi, over a period of two years (2010–2011). Stool samples were examined for the presence of parasites using direct, formal-ether concentration method and the Modified Ziehl Neelsen staining technique.

**Results:**

Overall, 541/2112 (25.6%) were positive for at least one intestinal parasite, with the common parasites being; *Entamoeba histolytica,* 225 (36.7%),*Cryptosporidium spp*. 187, (30.5%), *Giardia lamblia*, 98 (16%).The prevalence of intestinal parasites infection was higher among children from outpatient clinics 432/1577(27.4%) than among those admitted in hospital 109/535 (20.1%) p < 0.001. Infections with *E. histolytica*, and *G. lamblia* were higher among outpatients than inpatients (13.8% *vs* 1.3% p < 0.001 and 5.8% *vs* 1.3% p < 0.049) respectively, while infection with *Cryptosporidium spp*. was higher among inpatients than outpatients (15.3% *vs* 6.7%) respectively p < 0.001. Other parasites isolated among outpatients included *Isospora belli*, 19 (1.2%), *Ascaris lumbricoides,* 26 (1.6%), and *Hymenolepis nana* 12 (0.8%), with the remainder detected in less than ten samples each. HIV-infected participants were more likely to be infected with any parasite than uninfected participants, Adjusted Odds Ratio (AOR), 2.04, 95% CI, 1.55-2.67, p < 0.001), and with *Cryptosporidium spp*. (AOR, 2.96, 95% CI 2.07-4.21, p < 0.001).The inpatients were less likely to be infected with *E. histolytica* than outpatients (AOR, 0.11, 95% CI, 0.51- 0.24, p < 0.001), but more likely for inpatients to be infected with *Cryptosporidium spp*. than outpatients (AOR, 1.91, 95% CI, 1.33-2.73, p < 0.001). Mixed parasitic infections were seen in 65 (12.0%) of the 541 infected stool samples.

**Conclusion:**

Intestinal parasitic infections are common in urban informal settlements’ environment. Routine examinations of stool samples and treatment could benefit both the HIV infected and uninfected children in outpatient and inpatient settings.

## Background

Seventy three percent of deaths from diarrhoeal illness that occur among children in the developing world are concentrated in just 15 developing countries, including Kenya [1]. It is estimated that some 3.5 billion people are infected, and that 450 million are ill as a result of intestinal parasites and protozoan infections worldwide, the majority being preschool and school going children [2-4]. Since they commonly occur as mixed intestinal infections, they exacerbate concurrent immunosuppressive conditions such as malnutrition and HIV, and thus, contribute to poor health and impaired cognitive functions [5]. Children from resource-poor countries are more prone to intestinal and extra-intestinal parasitic diseases [6]. Children tend to be more active in the infected environment and rarely employ good sanitary behaviour [7]. Crowding amongst children for example in schools, orphanages and poor urban informal settlements, is likely to increase the opportunity for person-to-person transmission or environmental contamination with these parasites [8]. As a result, children who are the primary sufferers, may be an essential vector for the reintroduction of the pathogens to the local environment, hence maintain transmission [9].

Poverty as measured in terms of lack of sanitation, low literacy and overcrowding is associated with parasitic diseases in most communities [10]. In many low and middle-income countries urban migration has led to the creation of urban informal settlements with high rates of polyparasitism with both protozoa and helminths [11-16]. Overcrowding and poor sanitation in these areas lead to higher infection rates through closer proximity of the infected to larger vulnerable populations; parasite transmission thrives in these conditions [17]. Intestinal parasitic infections have enormous consequences on the health of HIV infected patients [18], and sub-Saharan Africa is already over burdened by HIV infection. These patients often suffer from frequent diarrhoeal episodes coupled with weight loss resulting from intestinal parasites, some of which can be fatal [19-22]. Concerted efforts in management of infections in HIV/AIDS patients have targeted diseases such as tuberculosis and other respiratory infections. Less attention has been given to emerging parasitic enteric infections, yet parasites such as *Cryptosporidium spp*, *Isospora belli*, *Microsporidia spp, Giardia intestinalis, Entamoeba spp*., *Cyclospora spp*., pose unique epidemiological constraints as they are ubiquitous in nature, treatment is still not yet available for some, and others are highly resistant to chlorination and other antiseptics [20]. Previous studies in Kenya have reported high prevalence of intestinal parasites ranging between 12.6% to 54% [23-26]. Polyparasitism was frequently observed in all these studies.To the best of our knowledge, there is no published data on the prevalence of intestinal parasitic infections in the informal settlements in Nairobi. Such data underlie decisions on the need for early diagnosis and management in order to prevent the health complications among vulnerable groups such as children, some of who could be HIV positive. In this study we examined the prevalence of intestinal parasitic infections among children aged 5 years and below, both HIV positive and HIV negative, living in Mukuru urban informal settlement, and either presenting at outpatient clinics, or admitted to the Mbagathi District hospital in Nairobi.

## Methods

### Study site and patients

This was a prospective cross sectional study of children aged 5 years and below who presented with diarrhoea to selected outpatient clinics (Reuben Centre, Lea toto and Medical Missionaries of Mary) which, are the only public dispensaries located in Mukuru informal settlement, as well as those admitted to the paediatric wards at Mbagathi District hospital from January 2010 to December 2011. Mukuru informal settlement is situated about 15 km east of the Nairobi city centre while Mbagathi District hospital is 5 km to the west of centre, and both serve patients from the slums and other low income areas from these locales. An episode of diarrhoea was defined as three or more loose bowel movements over a 24-hour period prior to the day of presentation. Basic demographic information including age, sex, and residence was collected on enrolment using a structured questionnaire. Consent was obtained from parents or guardians, after which counselling and testing for HIV was offered by qualified personnel as per the National guidelines [27]. Those children who tested HIV positive were referred to the comprehensive care centres within the study clinics and the Mbagathi District hospital for further management. The rainfall patterns for the study period, i.e. 2010–2011 were obtained from the Meteorological Department in Nairobi. The study was approved by the Kenya National Ethical Review Committee. All guardians of participating children were informed of the study objectives and voluntary consent was sought before inclusion. All laboratory results were included in the patients’ reports to the clinicians for appropriate management. The samples were examined for enteric parasitic infections, and were not analysed for viral or bacterial infections.

### Laboratory procedures

HIV testing was performed according to the Kenyan National Policy for all paediatric hospital admissions [27]. Specially formulated absorbent filter papers were used for collecting a drop of blood for infants under 18 months of age for molecular diagnosis through PCR [28]. Children aged 18 months or older were tested using two rapid antibody tests: Determine (Inverness Medical, USA) and Unigold (Trinity Biotech, Ireland).Fresh stool samples from children who met the inclusion criteria and enrolled in the study, were collected in clean polypots and labelled with a unique identifier. They were examined macroscopically for consistency, mucus, blood, and microscopically for the presence of ova, larvae, trophozoites and cysts of intestinal and extra-intestinal parasites. Stool examinations were performed by direct method and the formal-ether concentration techniques as described by Cheesbough [29]. Smears from the concentrates were stained using Modified Ziehl-Neelsen staining technique as described by Casemore [30], for identification of *Cryptosporidium spp.* and other protozoa.

### Data processing and analysis

Data from questionnaires was entered into a Microsoft Access database using EpiInfo™ version 3.3 [31]. Data cleaning procedures were performed before importing data for analysis into Stata 9.2 (Stata Corporation, Texas USA). Frequencies, proportions, medians and interquartile ranges (IQR) were used to describe the study population and parasitic infection. A Fisher’s exact chi-square test used to compare proportions. Non-parametric equality of medians test was used to compare medians. Logistic regression to identify potential correlates of infection with any parasite, and infection with each of the 3 most common parasites, and included all factors included in the unadjusted analysis in multivariate logistic regression models to determine significant and independent correlates of parasitic infection. Odds ratios were used to describe associations and a p-value of <0.05 was considered significant.

## Results

A total of 2,112 stool samples from children aged 5 years and below were collected for the study of which 1577 (75%) were from outpatient clinics in Mukuru informal settlements, and 535 (25%) were from children admitted to the paediatric ward at the Mbagathi District Hospital. Overall, 1116 (52.8%) were male and 996 (47.2%) were female. Sixteen children, who were eligible and were consented, did not give stool samples while 40 children were eligible but their parents/guardians declined to give consent. All were from the outpatient clinics.The median age of the children was 18 months (IQR: 9 to 32 months). Outpatients were older than inpatients: 24 months (IQR: 12 to 36 months) versus 10 months (IQR: 7 to 14 months), p < 0.001. On microscopic examination of stool samples 541 (25.6%) of all samples analysed were positive for at least one intestinal parasite. The prevalence of intestinal parasites was 613/1577 (38.9%), among children from outpatient clinics and 113/535 (21.1%) among those admitted in hospital. In total, fifteen types of parasites were isolated from children presenting at the outpatient clinics, with the three most common ones being *E. histolytica,* 218 (13.8%), *Cryptosporidium spp*. 105 (6.7%), *G.lamblia*, 91 (5.8%). Other parasites isolated include; *A.lumbricoides*, 26 (1.6%), *I.belli,*19 (1.2%), *H.nana* 12 (0.8%), while others including *C. mesnili, E. coli*, *H. diminuta*, *T. trichiura*, Taenia spp, hookworms, Cyclospora, *E. vermicularis* and *S. mansoni* were detected in less than 10 samples each, and contributed to 5% of all infections. Among inpatients, *Cryptosporidium spp*.,82/535 (15.3%) was the most common parasite, followed by I. belli, 16 (3.0%) while *G.lamblia* and *E. histolytica* were less common each with a prevalence of 1.3%, and Cyclospora which was detected in one sample (0.2%) (Table 1). Polyparasitism was observed in 65 (12.0%) of the 541 infected stool samples, details of this data are not shown.

**Table 1:** Patient characteristics by patient type, ( inpatient or outpatient) , for children with diarrhoea aged 5 years and below, recruited to a study of intestinal parasites in urban poor, Nairobi, between January 2010 and December 2011

**Patient characteristics**	**Total**	**(%)**	**Out-patient**	**(%)**	**In-patient**	**(%)**	**p-value**
Total	2112	100	1577	100	535	100	-
Sex							
Male	1116	52.8	813	(51.6)	303	(56.6)	0.045
Female	996	47.2	764	(48.4)	232	(43.4)	
Age group^1^							
0 to 12 months	758	36.4	402	(26.0)	356	(66.5)	<0.001*
13 to 24 months	528	25.3	387	(25.0)	141	(26.4)	0.564
25 to 36 months	408	19.6	377	(24.4)	31	(5.8)	<0.001*
37 to 48 months	241	11.6	237	(15.3)	4	(0.7)	<0.001*
49 to 60 months	148	7.1	145	(9.4)	3	(0.6)	<0.001*
Parasites infection							
*E. Hystolytica*	225	10.7	218	(13.8)	7	(1.3)	<0.001*
*Cryptosporidium spp.*	187	8.9	105	(6.7)	82	(15.3)	<0.001*
*G. lamblia*	98	4.6	91	(5.8)	7	(1.3)	<0.001*
*I. belli*	35	1.7	19	(1.2)	16	(3)	0.009*
*A. lumbricoides*	26	1.2	26	(1.6)	0	(0)	0.001*
*H. nana*	12	0.6	12	(0.8)	0	(0)	0.045*
*C. mesnili*	7	0.3	7	(0.4)	0	(0)	0.202
*E. coli*	6	0.3	6	(0.4)	0	(0)	0.347
*H. diminuta*	4	0.2	4	(0.3)	0	(0)	0.578
*T. trichiura*	3	0.1	3	(0.2)	0	(0)	0.576
*Taenia spp.*	3	0.1	3	(0.2)	0	(0)	0.576
*Hookworms*	2	0.1	2	(0.1)	0	(0)	1.000
*Cyclospora*	2	0.1	1	(0.1)	1	(0.2)	0.443
*E. vermicularis*	2	0.1	2	(0.1)	0	(0)	1.000
*S. mansoni*	1	0	1	(0.1)	0	(0)	1.000
Infected with any parasite							
Yes	541	(25.6)	432	(27.4)	109	(20.4)	
No	1571	(74.4)	1145	(72.6)	426	(79.6)	0.001*
HIV Status^2^							
Negative	1598	84.1	1226	(85.4)	372	(80.2)	0.009*
Positive	302	15.9	210	(14.6)	92	(19.8)	

KEY: *significant, missing values 1 = 29; 2 = 212.

### Associations of patient characteristics and infection with 3 common parasites

#### a) Entamoeba histolytica

In univariate analysis, gender, patient age, and patient type were individually associated with *E. histolytica* infection. Females were less likely to be infected (OR 0.74, 95% CI 0.56-0.98) p = 0.033. Children aged 13–24, 25–36, 37–48 and 49–60 months were more likely to be infected with *E. histolytica* compared to those up to 12 months old with 37–48 having highest odds (OR 1.86, 95% CI 1.22-2.84 p = 0.004; OR 3.0, 95% CI 1.98-4.53, p < 0.001; OR 4.02, 95% CI 2.57-6.29, p < 0.001; and OR 3.3, 95% CI 01.93-5.64, p = 0.033, respectively), (Table 2). Inpatients were less likely to be infected with *E. histolytica* than outpatients, (OR 0.83, 95% CI 0.04-0.18, p < 0.001), while there was no significant difference in *E. histolytica* infections between HIV positive and HIV negative children p = 0.225, (Table 2). In multivariate analysis, infection with *E. histolytica* infection was associated with gender, age and HIV status. Female children were more likely to be infected with *E. histolytica* infection than male children (AOR, 0.68; 95% CI 0.51-0.92, p = 0.012). Children older than 24 months were more likely to get the infection, compared to children aged up to 12 months (AOR, 2.04; 95% CI 1.31-3.19, p = 0.002), (AOR, 2.55; 95% CI 1.59-4.13, p < 0.001) and (AOR, 1.96; 95% CI 1.12- 3.43, p = 0.019) for age groups 25 to 36, 37 to 48 and 49 to 60 months, respectively. The HIV seropositive children were also more likely to get infected with E. histolytica than HIV seronegative children, (AOR, 1.58; 95% CI 1.18-2.33, p = 0.019). Inpatients were less likely to get the infection than outpatients (AOR, 0.11; 95% CI 0.51-0.24, p < 0.001), while there was no significant difference in these infections between wet and dry seasons (p = 0.769) (Table 2).

**Table 2:** Associations between patient characteristics and parasitic infection for children aged 5 years and below with diarrhoea in a study of intestinal parasites in urban poor, Nairobi, January 2010 to December 2011

**Patient characteristics**		***E. histolytica*(%)**	**Crude**			**Adjusted**		
			**OR**	**95% CI**	**p-value**	**AOR**	**95% CI**	**p-value**
Total		225/2112(10.7)	-	-	-	-	-	-
Gender								
Male (ref)		134/1116(12)	1.0	-	-	1.0	-	-
Female		91/996(9.1)	0.74	0.56,0.98	0.033*	0.68	0.51,0.92	0.012*
Age group^1^								
0 to 12 months (ref)		42/758(5.5)	1.0	-	-	1.0	-	-
13 to 24 months		52/528(9.8)	1.86	1.22,2.84	0.004*	1.46	0.93,2.28	0.098
25 to 36 months		61/408(15)	3.0	1.98,4.53	<0.001*	2.04	1.31,3.19	0.002*
37 to 48 months		46/241(19.1)	4.02	2.57,6.29	<0.001*	2.55	1.59,4.13	<0.001*
49 to 60 months		24/148(16.2)	3.3	1.93,5.64	<0.001*	1.96	1.12,3.43	0.019*
HIV status^2^								
Negative (ref)		178/1598(11.1)	1.0	-	-	1.0	-	-
Positive		41/302(13.6)	1.25	0.87,1.80	0.225	1.58	1.18,2.33	0.019*
Patient type								
Outpatients (ref)		218/1577(13.8)	1.0	-	-	1.0	-	-
Inpatients		7/535(1.3)	0.83	0.04,0.18	<0.001*	0.11	0.51,0.24	<0.001*
Seasonality				-	-			
Dry Season (ref)			1.0	-	-	1.0	-	-
Wet season	0		0.96	0.72,1.27	0.769	1.29	0.96,1.74	0.096

KEY: *significant, missing values 1 = 29; 2 = 212.

Infection with *Entamoeba histolytica.*

#### b) Cryptosporidium spp

**Figure 1:**
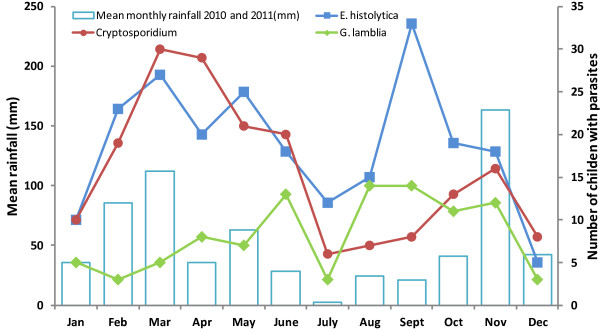
**Rainfall in proportion to monthly prevalence of *E. Histolytica, Cryptosporidium spp*. and *G. lamblia*.** The rainfall patterns in 2010–2011 as received from meteorological department were similar, hence the amounts indicated is the average of the two months in the two consecutive years.

For Cryptosporidiosis, univariate analysis did not show any significant differences in infection between males and females, p = 0.977, however, HIV seropositive children were three times more likely to be infected with *Cryptosporidium spp.* than the HIV seronegative ones (OR 3.07, 95% CI 2.19-4.32) p < 0.001. Age was also found to be significantly associated with *Cryptosporidium spp*. infection, whereby the prevalence was highest in children aged 13–24 months at 14.2%, this was the age group that was more likely to acquire cryptosporidiosis [(OR 1.49, CI, 1.06-2.09), p = 0.023]. Inpatients were equally more than twice at risk of cryptosporidium infection than outpatients [(OR 2.54, CI, 1.87-3.45), p < 0. 001]. In multivariate analysis, *Cryptosporidium spp*. infection was significantly associated with age groups 13 to 24 more likely to get the infection (AOR, 1.90, CI,1.31-2.75, p < 0.001), and 37 to 48 months group was inversely associated with Cryptosporidiosis (AOR, 0.39, 95% CI, 0.16-0.93, p = 0.034) as compared to children up to 12 months old. Positive HIV status and being an inpatient, was significantly associated with Cryptosporidiosis (AOR, 2.96, 95% CI, 2.07-4.21, p < 0.001) and (AOR; 1.91, 95% CI, 1.33-2.73, p < 0.001) respectively. Both univariate and multivariate analysis showed that cryptosporidiosis was seasonal with more infections occurring during wet the season [(OR 1.72, CI, 1.23-2.41), p < 0.001; AOR, 1.45, CI,1.01-2.09, p = 0.042] (Table 3, Figure 1).

**Table 3:** Associations between patient characteristics and parasitic infection for children aged 5 years and below with diarrhoea in a study of intestinal parasites in urban poor, Nairobi, January 2010 to December 2011

**Patient characteristics**		***Cryptosporidium spp*. (%)**	**Crude**			**Adjusted**		
			**OR**	**95%CI**	**p-value**	**AOR**	**95%CI**	**p-value**
Total		187/2112(8.9)	-	-	-	-	-	-
Gender								
Male (ref)		99/1116(8.9)	1.0	-	-	1.0	-	-
Female		88/996(8.8)	0.99	0.74,1.34	0.977	1.03	0.75,1.42	0.864
Age group^1^								
0 to 12 months (ref)		76/758(10)	1.0	-	-	1.0	-	-
13 to 24 months		75/528(14.2)	1.49	1.06,2.09	0.023*	1.90	1.31,2.75	0.001*
25 to 36 months		23/408(5.6)	0.54	0.33,0.87	0.011*	0.89	0.52,1.52	0.681
37 to 48 months		6/241(2.5)	0.23	0.98,0.53	0.001*	0.39	0.16,0.93	0.034*
49 to 60 months		6/148(4.1)	0.38	0.16,0.89	0.025*	0.59	0.24,1.40	0.245
HIV status^2^								
Negative (ref)		117/1598(7.3)	1.0	-	-	1.0	-	-
Positive		59/302(19.5)	3.07	2.19,4.32	<0.001*	2.96	2.07,4.21	<0.001*
Patient type								
Outpatients (ref)		105/1577(6.7)	1.0	-	-	1.0	-	-
Inpatients		82/535(15.3)	2.54	1.87,3.45	<0.001*	1.91	1.33,2.73	<0.001*
Seasonality				-	-			
Dry Season (ref)			1.0	-	-	1.0	-	-
Wet season	0		1.72	1.23,2.41	<0.001*	1.45	1.01,2.09	0.042*

KEY: *significant, missing values 1 = 29; 2 = 212.

Infection with *Cryptosporidium spp.*

#### c) Giardia lamblia

In unadjusted analysis, children in age group 13 to 24 months were approximately 3 times more likely to be infected with *G. lamblia* than children below 13 months old (OR, 3.41, 95% CI, 1.61-7.22, p = 0.001), while children in age group 25 to 36 months were 5 times more likely to get the infection than children below 13 months old (OR, 5.10, 95% CI, 2.43-10.67, p < 0.001). Infections with *G. lamblia*, just like with *E. histolytica* were highest in 37–48 months age group at 10.8%, and the children in this age group were more than twice as likely to get infected compared to those below 13 months old, (OR, 9.05, 95% CI, 4.30-19.05), p < 0.001). The odds for giardiasis among children between 49 and 60 months old was 7.20, 95% CI, 3.10-16.76, p < 0.001. Inpatients were less likely to acquire giardiasis than outpatients, [OR, 0.22, 95% CI, 0.10-0.47), p < 0.001)]. There were no significant associations between Giardia infections and gender (p = 0.383), and HIV status (p = 0.497). In adjusted analysis, giardiasis was not associated with gender (p = 0.193), and HIV status (p = 0.946) but was significantly associated with age groups 13–24, 25–36, 37–48 and 49–60 months compared with children up to 12 months old, (AOR, 2.36, 95% CI, 1.07-5.14, p = 0.033), (AOR, 3.26, 95% CI, 1.49-7.13, p = 0.003), (AOR, 5.86, 95% CI, 2.67-12.89, p < 0.001), (AOR, 3.95, 95% CI, 1.59-9.78, p < 0.003), respectively. Inpatients were less likely to get the infection than outpatients (AOR, 0.41, 95% CI, 0.17-1.00, p = 0.049), (Table 4).

**Table 4:** Associations between patient characteristics and parasitic infection for children aged 5 years and below with diarrhoea in a study of intestinal parasites in urban poor, Nairobi, January 2010 to December 2011

**Patient characteristics**		***Giardia lamblia (*%)**	**Crude**			**Adjusted**		
			**OR**	**95%CI**	**p-value**	**AOR**	**95%CI**	**p-value**
Total		98/2112(4.6)	-	-	-	-	-	-
Gender								
Male (ref)		56/1116(5)	1.0	-	-	1.0	-	-
Female		42/996(4.2)	0.83	0.55,1.26	0.383	0.74	0.48,1.16	0.193
Age group^1^								
0 to 12 months (ref)		10/758(1.3)	1.0	-	-	1.0	-	-
13 to 24 months		23/528(4.4)	3.41	1.61,7.22	0.001*	2.36	1.07,5.14	0.031*
25 to 36 months		26/408(6.4)	5.10	2.43,10.67	<0.001*	3.26	1.49,7.13	0.003*
37 to 48 months		26/241(10.8)	9.05	4.30,19.05	<0.001*	5.86	2.67,12.89	<0.001*
49 to 60 months		13/148(8.8)	7.20	3.10,16.76	<0.001*	3.95	1.59, 9.78	0.003*
HIV status^2^								
Negative (ref)		78/1598(4.9)	1.0	-	-	1.0	-	-
Positive		12/302(4)	0.81	0.43,1.50	0.497	1.02	0.54,1.93	0.946
Patient type								
Outpatients (ref)		91/1577(5.8)	1.0			1.0		
Inpatients		7/535(1.3)	0.22	0.10,0.47	<0.001*	0.41	0.17,1.00	0.049*
Seasonality				-	-			
Dry Season (ref)			1.0	-	-	1.0	-	-
Wet seas	0		0.60	0.40,0.91	<0.015	0.79	0.51,1.21	-0.280

KEY: *significant, missing values 1 = 29; 2 = 212.

Infection with *Giardia lamblia.*

### Associations of patient characteristics and infection with any parasite

Infection with any parasite was highest among children in age group 37–48 months (34.9%) among HIV-infected (36.8%), and outpatients (27.4%). In unadjusted analysis, age, HIV status and patient type were individually associated with parasite infection. Compared to children younger than 13 months, children in age groups 13–24, 25–36, 37–48 and 49–60 months had significantly higher infection with any parasite, (OR, 1.69, 95% CI, 1.29-2.20, p < 0.001), (OR, 1.87, 95% CI, 1.41-2.47, p < 0.001), (OR, 2.34, 95% CI, 1.70-3.23, p < 0.001), (OR, 1.91, 95% CI, 1.29-2.84, p < 0.001), respectively. Children who were HIV-infected were more likely to be infected with any parasite (OR, 1.75, 95% CI, 1.35-2.27, p < 0.001), while inpatients were less likely to get infected with any parasite than outpatients, (OR, 0.68, 95% CI, 0.53-0.86, p < 0.001), (Table 5). In adjusted analysis, age and HIV status remained significantly associated with parasite infection while patient type was not. Children in age groups 13–24, 25–36, 37–48 and 49–60 months had significantly higher infection with any parasite than children below 13 months old, (adjusted odds ratio (AOR), 1.72, 95% CI, 1.30-2.29, p < 0.001), (AOR, 2.01, 95% CI, 1.46-2.76, p < 0.001), (AOR, 2.53, 95% CI, 1.77-3.64, p < 0.001), and (AOR, 1.85, 95% CI, 1.20-2.85, p = 0.005), respectively. HIV-infected children were more likely to get infected with any parasite (AOR, 2.04, 95% CI, 1.55-2.67, p < 0.001). Infections with any parasite were significantly associated with wet season (AOR, 1.35, 95% CI, 1.09-1.68, p = 0.007) (Table 5).

**Table 5:** Associations between patient characteristics and infection with any parasite from a study of intestinal parasites in children aged in urban poor, Nairobi, 2010-2011

**Patient characteristics**	**Any parasite (%)**		**Crude**			**Adjusted**		
			**OR**	**95% CI**	**p-value**	**AOR**	**95% CI**	**p-value**
Total	541/2112(25.6)		-	-	-	-	-	-
Gender								
Male (ref)	298/1116(26.7)		1.0	-	-	1.0	-	-
Female	243/996(24.4)	0.89	0.73,1.08	0.226		0.86	070,1.06	0.169
Age group^1^								
0 to 12 months (ref)	141/758(18.6)	1.0	-	-		1.0	-	-
13 to 24 months	147/528(27.8)	1.69	1.29,2.20	0.001*		1.72	1.30,2.29	<0.001*
25 to 36 months	122/408(29.9)	1.87	1.41,2.47	<0.001*		2.01	1.46,2.76	<0.001*
37 to 48 months	84/241(34.9)	2.34	1.70,3.23	<0.001*		2.53	1.77,3.64	<0.001*
49 to 60 months	45/148(30.4)	1.91	1.29,2.84	0.001*		1.85	1.20,2.85	0.005*
HIV status^2^								
Negative (ref)	398/1598(24.9)	1.0	-	-		1.0	-	-
Positive	111/302(36.8)	1.75	1.35,2.27	<0.001*		2.04	1.55,2.67	<0.001*
Patient type								
Outpatients (ref)	432/1577(27.4)	1.0				1.0		
Inpatients	109/535(20.4)	0.68	0.53,0.86	0.001*		0.78	0.59,1.03	0.084
Seasonality Dry Season (ref) Wet season 0			-	-				
		1.0 1.19	- - 0.97, 1.46	- - 0.087		1.0 1.35	- 1.09,1.68	- 0.007*

KEY: *significant, missing values 1 = 29; 2 = 212.

### Prevalence of parasites by seasons

Data from the meteorological department, Nairobi, showed a bimodal distribution of rainfall during the study period with rainy season being experienced between February to May and September to December, with the wettest months being March and November, while the dry season was in June to August, and December to January. Frequencies of parasite infections followed this bimodal distribution with highest prevalence being observed between March and May and September to November, and least infections observed in July, which was the driest month (Figure 1).

## Discussion

In this cross-sectional study intestinal parasitic infections were frequent and high proportions of polyparasitism were reported, whereby infections with *E. histolytica/dispar*, and *G.lamblia* were higher among outpatients than inpatients(13.8% *vs* 1.3% and 5.8% *vs* 1.3%) respectively, while infection with *Cryptosporidium spp*. was higher among inpatients than outpatients (15.3% *vs* 6.7%) respectively. These were the most common enteric parasites associated with diarrhoea in the study population. This is in agreement with an earlier study carried out in Kenya where these three were the most commonly isolated parasites [32]. Higher prevalence has been reported among children in the urban slums of Karachi, Pakistan, where prevalence of 52.8% was reported [33] and also in children in urban Amman, Jordan, which was at 78% [34]. The high prevalence of intestinal parasitic infections in these urban slum settings is linked to lack of sanitation, lack of access to safe water and improper hygiene; therefore they occur wherever there is poverty. The prevalence of helminths reported in this study was lower than that of protozoa. This could be attributed to the age of the study population which was less than five years, as reported in other similar studies [35-37]. The intestinal parasitic infections were found to be associated with age with most infections occurring in children aged 24 months and above**,** and significantly increase with age, with children aged 37- 48 months (34.9%) more likely to present with intestinal parasitic infections, AOR,1.77,CI,1.77-3.64,p < 0.001, than other age groups. This has also been observed by Nyantekye [38] in children in Ethiopia, Moyo et al. [39] in Tanzania, as well as in Kenya by Thiongo et al. [40]. This could be either due to higher rates of transmission or accumulative parasites over time. In Kenya,1.5 million persons are living with HIV, 13.3% being children [41]. Intestinal parasitic infections are among the leading causes of mortality and morbidity among patients infected with HIV and, specifically, gastrointestinal protozoa which cause significant morbidity in children and are opportunistic infections in HIV/ AIDS patients [21,22,42]. In our study population 36.8% of HIV infected children had parasitic infections as compared to 24.9% HIV negative children, with adjusted odds of 2.04,CI,1.55-2.67,p < 0.001. Prevalence of intestinal parasitic infections among HIV infected patients were also found to be considerably higher in Malaysia, (37.9%), [43], Ethiopia (52.6% and 59.8%) [44,45] respectively, Nigeria (79.3%) [46] and Indonesia (84.3%) [22]. In addition, children admitted to hospital were less likely to be infected with intestinal parasites than those seen at the outpatient clinics (20.4% vs 27.4%, AOR, 0.68, CI, 0.59-1.03, p < 0.084). It is likely that most of the children admitted in hospital may have been treated elsewhere in outpatient clinics in the community before being admitted in hospital. Significant association between Cryptosporidiosis and HIV infection was evident with HIV positive children being at least three times more likely to be infected with *Cryptosporidium spp.* (19.5%) than the HIV negative ones (7.3%) (AOR, 2.96, 95% CI 2.07-4.21) p < 0.001. Higher findings have been reported in Uganda with prevalence of 73.6% in HIV infected children. The high percentages recorded in Uganda may be, due to the high sensitivity of the molecular techniques used in the studies, whereas the prevalence rates reported in our study are based on microscopy, which is less sensitive. The prevalence in HIV negative children (7.3%) was comparable to that reported in Uganda (5.9%) [47] and also in China (3.0%) [48]. Cryptosporidiosis was highest among children 13–24 months of age as compared to 0–12 months agegroup. This is in agreement with earlier reports in Kenyan children [32], and similar observations were made in a study in Gaza [49], but it is in contrast to reports on cryptosporidiosis in children in Egypt, where infection was most common among children less than 12 months of age [50,51]. It is not clear why there are differences in susceptibility to infection by age of children, but this may be due to the prevailing *Cryptosporidium spp.* endemic in specific areas. Understanding the transmission dynamics warrants further investigation of the *Cryptosporidium spp*. and subtypes and genotypes in circulation. Most intestinal parasitic infections were observed to be higher among the outpatients than inpatients except infections with *Cryptosporidium spp*., whose prevalence among outpatients was 6.7%, and this was twice as high in inpatients at 15.3% and the difference was statistically significant, (AOR, 1.91, 95% CI 1.33-2.73, p < 0.001). This might be attributed to the fact that *Cryptosporidium spp* is difficult to treat and does not respond to the commonly used antiprotozoal drugs except nitazoxanide [52]. This drug is not readily available especially in resource poor settings, hence the high prevalence of these infections among the inpatients than outpatients (15.3% vs 6.7%, p < 0.001), even though most of these children were already on treatment in the wards. *Giardia lamblia* and *E. Histolytica* infections increased significantly with age with the most affected age group being the 37–48 months, (10.8%, AOR, 5.86, CI, 2.67-12.89, p < 0.001 and 19.1%, AOR, 2.55, CI, 1.59-4.13, p < 0.001) respectively. Considering that these children live in slum areas where the sanitation is poor, with open sewers, and limited access to clean water, they normally play in the soil which harbours these parasites and are less mindful of important personal hygiene practices such as washing of hands with soap and water before eating, after playing and after visiting the toilets. *Giardia* cysts are highly resistant to environmental conditions, being able to survive in the environment for long periods of time, [53]. Acknowledging the resilience of these cysts, it is conceivable that protozoan infections are much more frequent in poor settings than estimated, even in the absence of reported outbreaks and epidemiological surveys [54]. *G.lamblia* was more common among outpatients than inpatients (5.8% and 1.3%) respectively. Similar observations have been made elsewhere in studies on urban children, though with higher prevalence observed [55-57]. Polyparasitism (duo and triple infections) was observed in 12% of the infected samples, mainly involving *E. Histolytica* with one or two other enteric parasites. This is common and widely reported in children elsewhere [58,59]. Seasonal variations are known to affect the prevalence of a number of infections. Although Tuli and colleagues [60] relate intestinal coccidian infection to seasonal variations, there is paucity of reports on effect of seasonal variations on the prevalence of intestinal parasitic infections. These infections showed bimodal transmission with the highest infection rates of observed during wet months. This has also been reported in Malawi where peak prevalence was observed during rainy season [61], as well as in Nigeria [62]. Rainy season may facilitate conditions and risk factors that predispose people to intestinal parasitic infections. The faeces are washed into nearby streams and open sewers that flow along the shanties in the overcrowded urban informal settlements, and can lead to contamination of drinking water, hence, increased infections and indeed, higher prevalence.

## Conclusion

In conclusion, the magnitude of intestinal parasitic infections among the study population of an urban informal settlement in Nairobi is high in children. Routine examination of stool samples for parasites, including Cryptosporidium could significantly benefit both the HIV infected and uninfected children by contributing to reduce morbidity and improved quality of life.

## Competing interests

The authors have declared that no competing interests exists.

## Authors’ contribution

CK,SK,NDJ conceived and designed the study protocol and questionnaires for interviews. CK, EM, NJ conducted interviews, performed data and stool collection and provided laboratory analyses of stool samples. AW and CK did the data analysis. Planning, coordination and supervision of data collection in the field, data entry and cleaning, and writing up of the manuscript was done by CK. SK,NDJ, and AW revised the manuscript. The final version of the manuscript was reviewed and approved by all authors prior to submission. All authors read and approved the final manuscript.

## Pre-publication history

The pre-publication history for this paper can be accessed here:

http://www.biomedcentral.com/1471-2334/13/243/prepub

## References

[B1] WHO/ UNICEF Diarrhoea: Why children are still dying and what can be done? 2009 WHO/ UNICEF Report 1 16

[B2] Montresor A Crompton DWT Bundy DAP Hall A Savioli L Guidelines for the evaluation of soil-transmitted helminthiasis and schistosomiasis at community level: A guide for managers of control programmes Trans R Soc Trop Med Hyg 1998 92 4 470 471

[B3] WHO Removing Obstacles to development. Report on Infectious diseases 1999 Geneva Switzerland: World Health Organization bulletin

[B4] Bethony J Brooker S Albonico M Geiger SM Loukas A Diemert D Hotez PJ Soil-transmitted helminth infections: ascariasis, trichuriasis, and hookworm Lancet 2006 367 1521 1532 10.1016/S0140-6736(06)68653-4 16679166

[B5] Amadi B Mwiya M Chomba E Thomson M Chintu C Kelly P Walker-Smith J Improved nutritional recovery on an elemental diet in Zambian children with persistent diarrhoea and malnutrition J Trop Pediatr 2005 51 5 10 10.1093/tropej/fmh064 15601655

[B6] Andersen PL Amebiasis Ugeskr Laeger 2000 162 11 1537 1541 10868107

[B7] Korkes F Kumagai FU Belfort RN Szejnfeld D Abud TG Kleinman A Florez GM Szejnfeld T Chieffi PP Relationship between Intestinal Parasitic Infection in Children and Soil Contamination in an Urban Slum J Trop Pediatr 2009 55 1 42 45 18499735 10.1093/tropej/fmn038

[B8] Alkhalife IS Retrospective analysis of intestinal parasitic infections diagnosed at a University Hospital in Central, Saudi Arabia Saudi Med J 2006 27 11 1714 1718 17106548

[B9] Ngui R Ishak S Chow SC Mahmud R Yvonne ALL Prevalence and Risk Factors of Intestinal Parasitism in Rural and Remote West Malaysia PLoS Negl Trop Dis 2011 5 3 1 7 10.1371/journal.pntd.0000974 PMC3046966 21390157

[B10] Albonico M Montresor A Crompton DW Savioli L Intervention for the control of soil-transmitted helminthiasis in the community Adv Parasitol 2006 61 311 48 16735168 10.1016/S0065-308X(05)61008-1 PMC5633078

[B11] Angeles G Lance P Barden-O’Fallon J Islam N Mahbub AQ Nazem NI The census and mapping of slums in Bangladesh: design, select results and application Int J Health Geogr 2009 8 32 38 10.1186/1476-072X-8-32 19505333 PMC2701942

[B12] Warunee N Choomanee L Sataporn P Rapeeporn Y Nuttapong W Sompong S Thongdee S Bang-on S Rachada K Intestinal parasitic infections among school children in Thailand Trop Biomed 2007 24 2 83 88 18209713

[B13] Al Kilan MK Dahesh SM El Taweel HA Intestinal parasitosis in Nalout popularity, western Libya J Egypt Soc Parasitol 2008 38 2 393 8 19143135

[B14] Mukherjee AK Chowdhury P Bhattacharya MK Ghosh M Rajendran K Ganguly S Hospitalbased surveillance of enteric parasites in Kolkata BMC Res. Notes 2009 2 110 113 10.1186/1756-0500-2-110 19545355 PMC2706841

[B15] Appleton CC Mosala TI Levin J Olsen A Geohelminth infection and re-infection after chemotherapy among slum-dwelling children in Durban Ann Trop Med Parasitol 2009 103 3 249 261 10.1179/136485909X398212 19341539

[B16] Mumtaz S Siddiqui H Ashfaq T Frequency and risk factors for intestinal parasitic infection in children under five years age at a tertiary care hospital in Karachi J Pak Med Assoc 2009 59 4 216 219 19402281

[B17] Brooker S Clements AC Bundy DA Global epidemiology, ecology and control of soil-transmitted helminth infections Adv Parasitol 2006 62 221 261 16647972 10.1016/S0065-308X(05)62007-6 PMC1976253

[B18] Nissapatorn V Lessons learned about opportunistic infections in southeast Asia Southeast Asian J Trop Med Public Health 2008 39 4 625 641 19058599

[B19] Okodua M Adeyeba OA Tatfeng YM Okpala HO Age and sex distribution of intestinal parasites infections among HIV infected subjects in Abeokuta Nigeria J Health Allied Sci 2003 4 3 5

[B20] Mariam ZT Abebe G Mulu A Opportunistic and other intestinal parasitic infections in AIDS patients, HIV seropositive healthy carriers and HIV seronegative individuals in Southwest Ethiopia E Afr J Pub Health 2008 5 3 169 172 19374319

[B21] Ajjampur SS Sankaran P Kang G Cryptosporidium species in HIV-infected individuals in India: an overview Natl Med J India 2008 21 4 178 184 19267039

[B22] Kurniawan A Karyadi T Dwintasari SW Sari IP Yunihastuti E Djauzi S Smith HV Intestinal parasitic infections in HIV/ AIDS patients presenting with diarrhoea in Jakarta, Indonesia Trans R Soc Trop Med Hyg 2009 103 9 892 898 10.1016/j.trstmh.2009.02.017 19327806

[B23] Chunge RN Nagelkerke N Karumba PN Kaleli N Wamwea M Mutiso N Andala EO Gachoya J Kiarie R Kinoti SN Longitudinal study of young children in Kenya: intestinal parasitic infection with special reference to Giardia lamblia, its prevalence, incidence and duration, and its association with diarrhoea and with other parasites Acta Trop 1991 50 1 39 49 10.1016/0001-706X(91)90071-Q 1686143

[B24] Joyce T McGuigan KG Elmore-Meegan M Conroy RM Prevalence of enteropathogens in stools of rural Maasai children under five years of age in the Maasailand region of the Kenyan Rift Valley East Afr Med J 1996 73 1 59 62 8625866

[B25] Saidi SM Lijima Y Sang WK Mwangudza AK Oundo JO Taga K AAihara M Nagayama K Yamahoto H Waiyaki PG Honda T Epidemiological study on infectious diarrhoeal diseases in children in a coastal rural area of Kenya Microbiol Immunol 1997 41 10 773 778 9403500 10.1111/j.1348-0421.1997.tb01925.x

[B26] Nyarango RM Peninah AA Ephantus WK Nyanchongi BO The risk of pathogenic intestinal parasite infections in Kisii Municipality, Kenya BMC Publ Health 2008 8 237 243 10.1186/1471-2458-8-237 PMC2478685 18620608

[B27] National AIDS and STI Control Programme (NASCOP) and Ministry of Public Health and Sanitation National guidelines for HIV Counselling and Testing in Kenya 2010 2 Kenya: NASCOP,MOPHS Pp. 13 14

[B28] Sherman G MD G Dried Blood Spots Improve Access to HIV Diagnosis and Care for Infants in Low-Resource Settings JAIDS Journal of Acquired Immune Deficiency Syndromes 2005 5 615 617 10.1097/01.qai.0000143604.71857.5d 15793374

[B29] Cheesbrough M District Laboratory Practice in Tropical Countries. Part 2 2004 Cambridge, UK: Cambridge University Press 299 329

[B30] Casemore DP Laboratory methods for diagnosing cryptosporidiosis. Broadsheet 128 J Clin Pathol 1991 44 445 451 10.1136/jcp.44.6.445 1712367 PMC496821

[B31] Centres for Disease Control (CDC,USA) Epi Info™ 200423716816

[B32] Gatei W Wamae CN Mbae C Waruru A Mulinge E Waithera T Gatika SM Kamwati SK Revathi G Hart CA Cryptosporidiosis: prevalence, genotype analysis, and symptom associated with infections in children in Kenya Am J Trop Med Hyg 2006 75 78 82 16837712

[B33] Mehraj V Hatcher J Akhtar S Rafique G Beg MA Prevalence and Factors Associated with Intestinal Parasitic Infection among Children in an Urban Slum of Karachi PLoS One 2008 3 11 1 6 10.1371/journal.pone.0003680 PMC2577067 18997865

[B34] Shakkoury WA Wandy EA Prevalence of Giardia lamblia infection in Amman, Jordan Pak J Med Sci 2005 21 2 199 201

[B35] Muniz PT Ferreira MU Ferreira CS Conde WL Monteiro CA Intestinal parasitic infections in young children in São Paulo, Brazil: prevalences, temporal trends and associations with physical growth Ann Trop Med Parasitol 2002 96 5 503 512 10.1179/000349802125001311 12194711

[B36] Pullan RL Bethony JM Geiger SM Correa-Oliveira R Brooker S Quinnell RJ Human helminth co-infection: no evidence of common genetic control of hookworm and *Schistosoma mansoni* infection intensity in a Brazilian community Int J Parasitol 2010 40 3 299 306 10.1016/j.ijpara.2009.08.002 19699204 PMC2824627

[B37] Bethony J Chen J Lin S Xiao S Zhan B Li S Xue H Xing F Humphries D Yan W Chen G Foster V Hawdon JM Hotez PJ Emerging Patterns of Hookworm Infection: Influence of Aging on the Intensity of *Necator* Infection in Hainan Province, People‘s Republic of China Clin Infect Dis 2002 35 11 1336 1344 10.1086/344268 12439796

[B38] Nyantekyi LA Legesse M Belay M Tadesse K Tadesse K Manaye K Macias C Erko B Intestinal parasitic infections among under-five children and maternal awareness about the infections in Shesha Kekele, Wondo Genet, Southern Ethiopia Ethiop J Heal Dev 2011 186 190

[B39] Moyo SJ Gro N Matee MI Kitundu J Myrmel H Mylvaganam H Maselle SY Langeland N Age specific aetiological agents of diarrhoea in hospitalized children aged less than five years in Dar es Salaam, Tanzania BMC Pediatr 2011 23 11 19 25 21345186 10.1186/1471-2431-11-19 PMC3050719

[B40] Thiongo J Mucheru O Muite F Langat B Kamau P Ireri L Spatial Distribution of *Giardia intestinalis* in Children up to 5 Years Old Attending Out-patient Clinic at Provincial General Hospital, Embu, Kenya Res J Parasitol 2011 6 136 143 10.3923/jp.2011.136.143

[B41] UNAIDS The Kenya AIDS epidemic Update. AIDSINFO, Epidemic update and health sector progress towards Universal Access Joint United Nations Programme on HIV/AIDS (UNAIDS) and World Health Organization (WHO) 2011 pp12 26

[B42] Eza D Cerrillo G Moore DA Postmortem findings and opportunistic infections in HIV-positive patients from a public hospital in Peru Pathol Res Pract 2006 202 767 775 10.1016/j.prp.2006.07.005 16979302 PMC2912516

[B43] Asma I Johari S Benedict LH Benedict LHS AL L How common is intestinal parasitism in HIV-infected patients in Malaysia? Trop Biomed 2011 28 2 400 410 22041762

[B44] Getachew H Afework K Gemeda E Demekech D Endris M Fusao O Intestinal Parasitic Infections in HIV/AIDS and HIV Seronegative Individuals in a Teaching Hospital Ethiopia Jpn J Infect Dis 2004 57 41 43 15118206

[B45] Assefa S Erko B Medhin G Assefa Z Shimelis T Intestinal parasitic infections in relation to HIV status, diarrhoea and CD4 T-Cell count BMC Infecti Dis 2009 18 9 155 10.1186/1471-2334-9-155 PMC2751775 19765310

[B46] Adesiji YO Lawal RO Taiwo SS Fayemiwo SA Adeyeba OA Cryptosporidiosis in HIV infected patients with diarrhoea in Osun State southwestern Nigeria Eur J Gen Med 2007 4 119 122

[B47] Tumwine JK Kekitiinwa A Bakeera-Kitaka S Ndeezi G Downing R Feng X Akiyoshi DE Tzipori S Cryptosporidiosis and microsporidiosis in Ugandan children with persistent diarrhoea with and without concurrent infection with the human immunodeficiency virus Am J Trop Med Hyg 2005 73 921 925 16282304

[B48] Chong ZXX Survey on coinfection with HIV and intestinal parasites in high prevalence of HIV/AIDS, China Bing Fang Zhi Za Shi 2012 24 2 168 172 22799161

[B49] Sallon S El-Shawwa R Khalil M Ginsburg G El Tayib J El-Eila J Green V Hart CA Diarrhoeal disease in children in Gaza Ann Trop Med Parasitol 1994 88 175 182 8067813 10.1080/00034983.1994.11812856

[B50] Abdel-Messih IA Wierzba TF Abu-Elyazeed R Ibrahim AF Ahmed SF Kamal K Sanders J Frenck R Diarrhoea associated with Cryptosporidium parvum among young children of the Nile River Delta in Egypt J Trop Pediatr 2005 51 3 154 159 10.1093/tropej/fmh105 15831665

[B51] Cegielski JP Ortega YR McKee S Madden JF Gaido L Schwartz DA Manji K Jorgensen AF Miller SE Pulipaka UP Msengi AE Mwakyusa DH Sterling CR Reller LB Cryptosporidium, Enterocytozoon, and Cyclospora infections in pediatric and adult patients with diarrhoea in Tanzania Clin Infect Dis 1999 28 314 321 10.1086/515131 10064250

[B52] Cabada MM Clinton White A Jr Treatment of cryptosporidiosis: do we know what we think we know? Curr Opin Infect Dis 2010 23 494 499 10.1097/QCO.0b013e32833de052 20689422

[B53] Wallis PM Erlandsen SL Isaac-Renton JL Olson ME Robertson WJ Van Keulen H Prevalence of Giardia cysts and Cryptosporidium oocysts and characterization of Giardia spp. isolated from drinking water in Canada Appl Environ Microbiol 1996 62 8 2789 2797 8702271 10.1128/aem.62.8.2789-2797.1996 PMC168064

[B54] Escobedo AA Almirall P Alfonso M Cimerman S Rey S Terry SL Treatment of intestinal protozoan infections in children Arch Dis Child 2009 94 6 478 482 10.1136/adc.2008.151852 19329448

[B55] Fernandez MC Verghese S Bhuvaneswari R Elizabeth SJ Mathew T Anitha A Chitra AK A comparative study of the intestinal parasites prevalent among children living in rural and urban settings in and around Chennai J Commun Dis 2002 34 1 35 39 12718339

[B56] Rayan P Verghese S McDonnel PA Geographical locations and age affects the incidence of parasitic infestations in school children Ind 2010 53 3 498 502 10.4103/0377-4929.68292 20699511

[B57] Siwila J Isaac P Heidi Larsen E Mbiko N Annette O Seasonal prevalence and incidence of Cryptosporidium spp. and Giardia duodenalis and associated diarrhoea in children attending pre-school in Kafue, Zambia Trans R Soc Trop Med Hyg 2011 105 102 108 10.1016/j.trstmh.2010.10.004 21093003

[B58] Chia-Kwung F Chien-Wei L Shu-Yu L Hosea S Da-Der J Chia-Mei C Jien-Yu J Ying-Chieh H Peter Wu-Shou C Wen-Ta C Takeshi N Akiko T Ya-Hsin H Chi-Chen T Lan S-JJ Jane Chen-Jui C Jane Chen-Jui C Prevalence of intestinal parasitic infections among primary schoolchildren in areas devoid of sanitation in northwestern Kingdom of Swaziland, Southern Africapathogens and global health 2012 106 60 62 10.1179/2047773211Y.0000000017 22595276 PMC4001513

[B59] Lee KJ Ahn YK Yong TS A small-scale survey of intestinal parasite infections among children and adolescents in Legaspi city, the Philippines Korean J Parasitol 2000 38 183 185 10.3347/kjp.2000.38.3.183 11002656 PMC2721200

[B60] Tuli L Gulati AK Sundar S Mohapatra TM Correlation between CD4 counts of HIV patients and enteric protozoan in different seasons – An experience of the tertiary care hospital in Varanasi (India) Biomed Centr Gastroenterol 2008 8 36 10.1186/1471-230X-8-36 PMC2536662 18713475

[B61] Peng MM Wilson ML Holland RE Meshnick SR Lal AA Xiao L Genetic diversity of Cryptosporidium spp. in cattle in Michigan: implications for understanding the transmission dynamics Parasitol Res 2003 90 175 180 12783304 10.1007/s00436-003-0834-5

[B62] Akinbo FO Okaka CE Omoregie R Seasonal Variation of Intestinal Parasitic Infections Among HIV-Positive Patients in Benin City, Nigeria Ethiop J Health Sci 2011 21 3 191 194 22434999 PMC3275870

